# Safety, Tolerability, and Pharmacokinetics of Oral Ferric Maltol in Children With Iron Deficiency: Phase 1 Study

**DOI:** 10.1097/PG9.0000000000000090

**Published:** 2021-06-15

**Authors:** Stephen Allen, Marcus Karl-Heinz Auth, Jon Jin Kim, Babu Vadamalayan

**Affiliations:** From the * Department of Clinical Sciences, Liverpool School of Tropical Medicine, Liverpool, UK; † Department of Paediatric Gastroenterology, Hepatology and Nutrition, Alder Hey Children’s NHS Foundation Trust, Liverpool, UK; ‡ Faculty of Health and Life Sciences, University of Liverpool, Liverpool, UK; § Department of Paediatric Nephrology, Nottingham Children’s Hospital, Nottingham, UK; ∥ Paediatric Gastroenterology and Nutrition Service, King’s College Hospital NHS Foundation Trust, London, UK

**Keywords:** anemia, children, iron deficiency, iron-replacement therapy

## Abstract

Iron deficiency is common in children and can have negative effects on behavior and function. Standard oral ferrous iron replacement is poorly absorbed and can cause treatment-limiting gastrointestinal adverse events (AEs). Ferric maltol is formulated to improve gastrointestinal absorption and tolerability versus oral ferrous compounds. In adult phase 3 trials, it increased hemoglobin and iron stores versus placebo, with a gastrointestinal AE profile similar to placebo. Here, we assess different doses of ferric maltol in children with iron deficiency.

**Methods:**

This phase 1 trial involved children of age 10 to 17 years with ferritin <30 µg/L (or <50 µg/L with transferrin saturation [TSAT] <20%). Children were randomized 1:1:1 to oral ferric maltol 7.8 mg, 16.6 mg, or 30 mg twice daily for 9 days and once on day 10. The primary outcomes were iron uptake measures (serum iron and TSAT) and population pharmacokinetic analyses.

**Results:**

The trial included 37 children (mean age 14.0 years; baseline mean ± standard deviation ferritin 16.3 ± 8.02 µg/L). Ferric maltol increased iron uptake nondose-proportionally: serum iron and TSAT plateaued between the 2 higher doses on day 1 and were comparable across all doses on day 10. Twenty children (54%) experienced AEs (all mild/moderate, gastrointestinal 32%), with similar frequencies in each group.

**Conclusions:**

All 3 ferric maltol doses increased iron uptake in children with iron deficiency, even over the short study duration, and were well tolerated. Nondose-dependent changes in serum iron and TSAT indicate physiologic regulation of iron uptake to meet the body’s needs.

## Abstract 2

Supplemental Digital Content is available in the text.

**What Is Known**

- Iron deficiency is common in children and can profoundly impair energy levels, motor skills, behavior, and cognitive function.
- Ferric maltol is an oral iron replacement therapy clinically proven in adults with iron deficiency with or without anemia.

**What Is New**

- This phase 1 trial provides evidence that ferric maltol was well tolerated and increased iron uptake in children with iron deficiency, even over the short study duration of 10 days.
- Nondose-dependent changes in measures of iron uptake (serum ferritin and transferrin saturation) indicate physiologic regulation of iron uptake to meet the body’s needs.

## Introduction

Iron deficiency is common in children and, particularly if it progresses to anemia, can profoundly impair energy levels, motor skills, behavior, and cognitive function (1–6). Effective management of iron deficiency to increase hemoglobin concentrations to age-appropriate reference ranges and to replenish iron stores (7,8) is crucial for the child’s long-term well-being. Mild iron deficiency can be corrected by consumption of iron-rich food, avoidance of factors that inhibit iron absorption, such as milk and carbonated drinks, or concomitant ingestion of vitamin C (9).

When dietary changes are insufficient to correct iron deficiency, oral iron preparations are the mainstay of treatment, with recommended daily doses of elemental iron of 3 to 6 mg/kg (maximum daily dose 200 mg) depending on symptom severity, ferritin concentration, and patient age (10). Ferrous iron (Fe^2+^) compounds (sulfate, fumarate, and gluconate) in solid and liquid forms are widely available (8,11–13). However, particularly at the maximum dose and in tablet form (14,15), iron from these formulations may be poorly absorbed. Unabsorbed iron undergoes oxidation in the gut lumen and mucosa, propagating reactive oxygen species that can damage the intestine and cause potentially severe gastrointestinal adverse events (AEs), such as nausea, epigastric discomfort, and constipation (11,16–20). In addition, free iron in the colon may have adverse effects on the gut microbiome, increasing pathogen abundance and causing intestinal inflammation (21,22). Poor gastrointestinal tolerance can reduce compliance, impeding effective correction of iron deficiency (20,23–25).

In adults, intravenous iron is the standard of care for patients who are unable to tolerate oral ferrous iron compounds or if the degree of anemia warrants acute therapy (8). However, intravenous iron is considered less often in children for several reasons, including a paucity of safety data (1), the risk of iron overload and associated toxicities, and problematic intravenous access (1,8,26,27). There is thus a need for an alternative to intravenous iron therapy to treat iron deficiency in children, particularly those unable to tolerate oral ferrous iron compounds.

**FIGURE 1.**
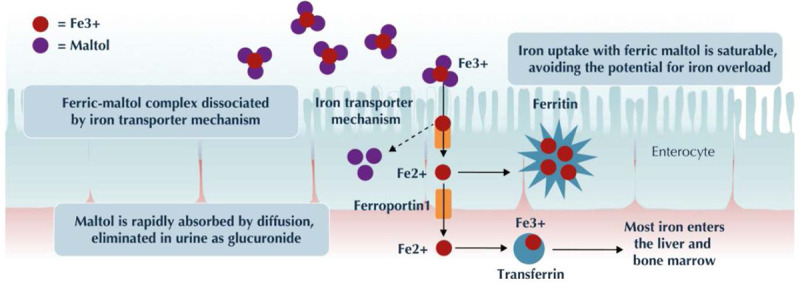
Ferric maltol mechanism of action. Fe2+ = ferrous iron; Fe3+ = ferric iron.

Ferric maltol is an oral iron-replacement therapy formulated to improve gastrointestinal absorption and tolerability compared with oral ferrous compounds (Fig. 1) (28–32). It contains ferric iron (Fe^3+^) tightly bound to maltol (3-hydroxy-2-methyl-4-pyrone), a naturally occurring sugar derivative that is widely used as a food additive (33,34). Maltol has a high affinity and selectivity for iron, providing a stable platform to deliver ferric iron to the intestine, where the higher affinity of ferric iron for the iron transporter mechanism promotes dissociation. Thus, iron is either taken up into the enterocytes when needed, via physiologic regulatory mechanisms, or eliminated as an intact complex with maltol in the feces, thus minimizing the amount of unbound iron forming free radicals in the gut and reducing the risk of gastrointestinal AEs. In addition, the ease with which maltol donates iron to the iron transporter mechanism at the point of absorption allows the iron to be taken up more efficiently than from ferrous formulations, and so the amount of elemental iron in each dose can remain relatively low (60 mg/day in adults), further minimizing the risk of AEs (19,20,28,29,31–33).

Ferric maltol has proven efficacy in phase 3 placebo-controlled trials involving adults with iron-deficiency anemia, including those with inflammatory bowel disease (IBD) (19,20) and chronic kidney disease (CKD) (35). In patients with IBD, ferric maltol significantly increased hemoglobin concentrations compared with placebo over 12 weeks (19) and maintained these improvements for up to 64 weeks (20). Similarly in patients with CKD, ferric maltol achieved statistical and clinically significant increases in hemoglobin and iron indices over 16 weeks (35). It is also associated with a low incidence of treatment-related gastrointestinal AEs in adults, with an event rate similar to that seen with placebo over 12 weeks in patients with IBD (19), a lower rate of toxicity-related treatment discontinuations than placebo in patients with CKD (35), and no evidence of accumulating toxicities with longer-term use (20). Here, we report data from a phase 1 study to assess the pharmacokinetics, safety, tolerability, and change in serum iron status of different doses of ferric maltol in children with iron deficiency.

## Methods

This phase 1, randomized, open-label, parallel-group pharmacokinetic study involved children seen as outpatients at 6 centers in the UK: Alder Hey Children’s NHS Foundation Trust; King’s College Hospital NHS Foundation Trust; University Hospitals of Leicester NHS Trust; Nottingham University Hospitals NHS Trust; Manchester University NHS Foundation Trust; and University College London Hospitals NHS Foundation Trust. The trial was conducted in accordance with the Declaration of Helsinki and with the consent of the relevant institutional ethics committees. Children aged 16 years or older and parents or guardians of children younger than 16 years provided written informed consent to participate before study initiation. The trial is registered with ClinicalTrials.gov under the identifier NCT03181451.

Children aged 10–17 years with confirmed iron deficiency at screening (defined as either ferritin <30 µg/L or ferritin <50 µg/L with transferrin saturation [TSAT] <20%) were eligible for inclusion in the study. Patients with or without anemia could be enrolled, provided hemoglobin was ≥8.5 g/dL at screening. Girls of childbearing potential had to agree to use adequate contraception during and until 4 weeks after the end of the study.

Children were excluded from the study if they had untreated or untreatable severe malabsorption syndrome (e.g. untreated celiac disease), concomitant disease that would compromise iron absorption and utilization (e.g., swallowing disorders or extensive small-bowel resection), extensive active bleeding (other than menstrual cycle), chronic renal disease (estimated glomerular filtration rate <30 mL/min), impaired liver function (alanine transaminase or aspartate transaminase >2 times upper limit of normal), active acute inflammatory disease, life-limiting illness, or any disease that, in the opinion of the investigator, could adversely affect the child’s safety. Pregnant or breastfeeding girls were also excluded, as were children who had participated in any other interventional clinical study within 28 days before screening, those who were scheduled to be hospitalized during the study period, and those with a known contraindication to iron preparations (e.g., hemochromatosis, chronic hemolytic disease, sideroblastic anemia, thalassemia, or lead intoxication-induced anemia) or hypersensitivity or allergy to ferric maltol or excipients used in the capsules.

The study comprised a screening period followed by a 10-day randomized treatment period and a post-treatment safety follow-up. Children were randomized 1:1:1 (stratified by age [10–14 vs 15–17 years] and sex) to ferric maltol 7.8 mg, 16.6 mg, or 30 mg twice daily (b.d.) for 9 days and once in the morning of day 10. The first and last doses of ferric maltol were taken in the presence of the investigator. Children took each dose with a glass of water 1 hour before or 2 hours after a meal. Doses were based on daily elemental iron requirements for the broad range of body weights of participants likely to be enrolled, with the aim of finding a minimum effective dose in this age group. The 30 mg b.d. adult dosage was chosen as the highest exposure; dosages of 16.6 mg b.d. and 7.8 mg b.d. were chosen as approximately one-half and one-quarter of the adult dose, respectively, with the exact dose depending on the full fill of available capsule shell sizes.

Children were not permitted to take oral or intravenous iron supplements or erythropoiesis-stimulating agents in the 28 days before screening and during the study, or to have blood transfusions within 12 weeks before screening and during the study. Concomitant antibiotics were not permitted at screening and during the study. Stable doses of other concomitant medications (unless possibly contributing to the patient’s anemia), vitamins, and supplements were permitted.

No formal sample size calculation was done for this study. On the basis of published reports, a population of 36 patients (12 per treatment group) was considered to be adequate to characterize iron uptake and pharmacokinetic parameters for the range of ferric maltol doses in an iron-deficient adolescent population (with or without anemia). All statistical analyses were performed using SAS version 9.3 or later (SAS Institute, Inc, Cary, NC).

The primary objectives were to assess iron uptake and ferric maltol pharmacokinetics. Serum iron and TSAT provided measures of iron uptake. In adults, dissociated maltol undergoes rapid glucuronidation and is cleared in urine as maltol glucuronide (36); therefore, in this study, plasma maltol glucuronide was measured to confirm the rate of clearance in children. The secondary objective was to assess the safety and tolerability of ferric maltol in terms of vital signs, AEs, concomitant medications, 12-lead electrocardiograms, and clinical laboratory blood tests.

Sparse pharmacokinetic blood sampling was performed at baseline before the morning dose and at prespecified collection windows 0.5–6 hours after the morning dose on days 1 and 10, giving 3 to 6 samples per time point. The post-dose pharmacokinetic sample collection windows were 0.5–<1, 1–<2, 2–<3, 3–<4, and 4–<6 hours, with patients assigned to post-dose pharmacokinetic blood sampling schedules in sequential order at randomization. Individuals had the same schedule on days 1 and 10. Plasma maltol glucuronide and serum iron and TSAT were measured at each time point to calculate standard pharmacokinetic parameters.

The intention-to-treat (ITT) population consisted of all patients who received at least 1 dose of study medication and had at least 1 evaluable post-dose pharmacokinetic sample. The observed values for plasma maltol glucuronide and serum iron and TSAT were summarized by dose group and visit for the ITT population, as were changes from baseline in serum iron and TSAT. The ITT population was also used in the population pharmacokinetic analysis. A base model was constructed by fitting various 1- and 2-compartment linear and nonlinear models to observed maltol glucuronide, iron, and TSAT concentrations using NONMEM (Nonlinear Mixed Effects Modelling) software (Icon plc, Dublin, Ireland) to find the model of best fit. This was used to test a series of covariates (age, race, and ethnicity, sex, current medical conditions, medical history relevant to iron-deficiency diagnosis, clinically significant medical history from the past 5 years including malignancies, sterilizations, hospitalizations, and surgeries, method of contraception for girls of childbearing potential, and body weight) to find covariates that influenced the base model to arrive at the final model. Concentrations–time profiles of plasma maltol glucuronide and serum iron and TSAT on days 1 and 10 were predicted for each patient using individual pharmacokinetic parameters and standard noncompartmental methods. The linear-up/log-down method (equivalent to the Linear Up/Log Down option in WinNonlin Professional, Certara, Princeton, NJ) was used in the computation of the area under the concentration–time curve (AUC). To explore dose proportionality, actual and dose-normalized maximum plasma concentration (C_max_) and AUCs were graphically displayed for each variable as functions of dose.

Safety analyses were conducted in all patients who received at least 1 dose of the study medication and had at least 1 subsequent visit (the safety population). Treatment-emergent adverse events (TEAEs) and serious adverse events (SAEs) were coded by system organ class and preferred term using the Medical Dictionary for Regulatory Activities version 19.0. Clinically significant changes from baseline in vital signs, physical examination, and routine clinical laboratory abnormalities were also recorded.

## Results

Forty-four children were screened: 5 failed screening because they did not meet inclusion/exclusion criteria, 1 was withdrawn by their parent/guardian during the screening period, and 1 was withdrawn before dosing because of the use of prohibited medication (Supplemental Fig. S1, Supplemental Digital Content, http://links.lww.com/PG9/A48). The remaining 37 children were randomized to ferric maltol 7.8 mg b.d. (n = 12), 16.6 mg b.d. (n = 13), and 30 mg b.d. (n = 12) and comprised both the ITT and safety populations (Supplemental Table S1, Supplemental Digital Content, http://links.lww.com/PG9/A48).

The groups were similar with respect to demographic and baseline characteristics (Table 1). The mean age of the overall population was 14.0 years (62% were aged 10–14 years) and the majority were white (65%) and female (65%). At baseline, the mean ± standard deviation (SD) ferritin concentration was 16.3 ± 8.02 µg/L (laboratory normal range: girls 13–150 μg/L, boys 30–400 μg/L) and hemoglobin concentration was 12.47 ± 1.12 g/dL (normal range: all children of age 6–11 years 11.5–15.5 g/dL, girls of age 12–17 years 12–16 g/dL, boys of age 12–17 years 13–16 g/dL). Most children were taking concomitant medications (84% overall, 83% in the 7.8 mg group, 100% in the 16.6 mg group, and 67% in the 30 mg group). Reflecting the most frequent comorbidities (especially Crohn disease, other gastrointestinal disorders, and headache), the most frequently used medications were adalimumab, azathioprine, mesalazine, omeprazole, and paracetamol.

**TABLE 1.** Participant Baseline Characteristics

	Ferric Maltol 7.8 mg b.d. (n = 12)	Ferric Maltol 16.6 mg b.d. (n = 13)	Ferric Maltol 30 mg b.d. (n = 12)	Total (N = 37)
Age, years				
Mean ± SD	14.1 ± 1.6	13.7 ± 1.8	14.2 ± 2.1	14.0 ± 1.8
Age group, n (%)				
10–14 years	8 (67)	7 (54)	8 (67)	23 (62)
15–17 years	4 (33)	6 (46)	4 (33)	14 (38)
Sex, n (%)				
Male	4 (33)	5 (39)	4 (33)	13 (35)
Female	8 (67)	8 (62)	8 (67)	24 (65)
Race,* n (%)				
Asian	2 (17)	3 (23)	2 (17)	7 (19)
Black or African American	1 (8)	2 (15)	2 (17)	5 (14)
White	9 (75)	8 (62)	7 (58)	24 (65)
Other	0 (0)	1 (8)	2 (17)	3 (8)
Unknown	1 (8)	0 (0)	0 (0)	1 (3)
Medical history,† n (%)				
Crohn disease	2 (17)	2 (15)	4 (33)	8 (22)
Vitamin D deficiency	3 (25)	2 (15)	2 (17)	7 (19)
Constipation	3 (25)	1 (8)	2 (17)	6 (16)
Abdominal pain	2 (17)	1 (8)	2 (17)	5 (14)
Headache	2 (17)	1 (8)	1 (8)	4 (11)
Chronic kidney disease	3 (25)	1 (8)	0	4 (11)
BMI, kg/m^2^				
Mean ± SD	22.7 ± 7.4	24.3 ± 7.8^‡^	19.8 ± 2.7	22.2 ± 6.4
Hemoglobin, g/dL				
Mean ± SD	12.3 ± 0.9	12.8 ± 1.1	12.4 ± 1.4	12.5 ± 1.1
Ferritin, µg/L				
Mean ± SD	16.2 ± 7.2	18.8 ± 8.3	13.8 ± 8.3	16.3 ± 8.

Baseline was defined as the last value observed before the first dose.

*Patients could record more than 1 race.

†Medical history disorders were reported in >10% of patients overall.

‡BMI was not measured in 2 children receiving ferric maltol 16.6 mg b.d.

b.d. = twice daily; BMI = body mass index; SD = standard deviation.

The mean number of days of study drug exposure was 9.9 for the 7.8 mg dose group, 8.8 for the 16.6 mg dose group, and 10.1 for the 30 mg dose group. Mean study drug compliance was 98%, 86%, and 98% in the 7.8 mg, 16.6 mg, and 30 mg dose groups, respectively.

**FIGURE 2.**
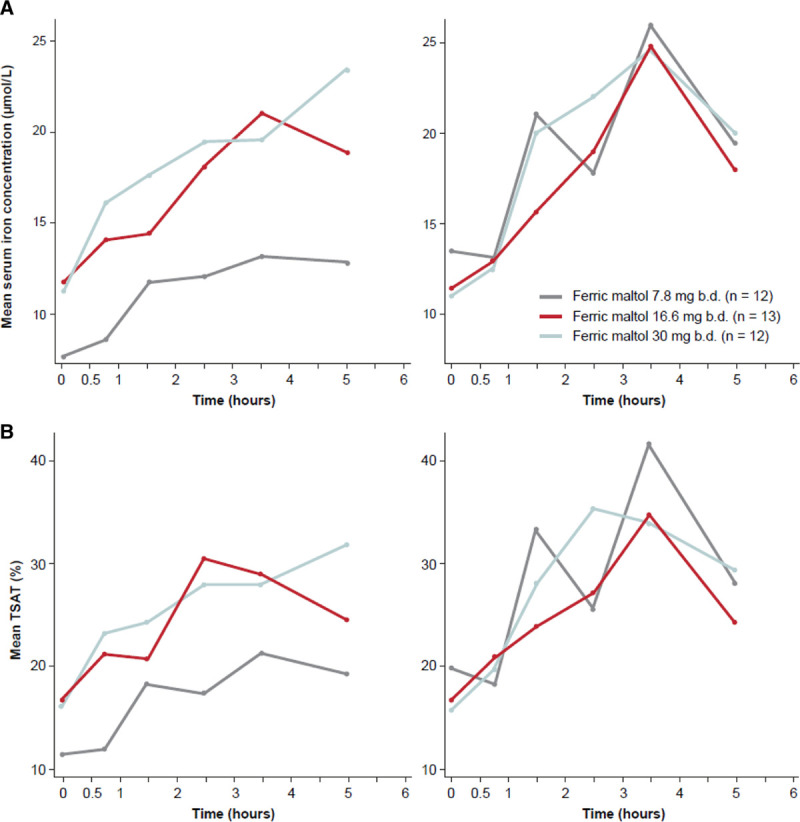
Response–time profiles for (A) mean serum iron (g/mL) and (B) mean TSAT (%) by ferric maltol dose on day 1 (left panels) and day 10 (right panels) in the intention-to-treat population. b.d. = twice daily; TSAT = transferrin saturation.

Administration of ferric maltol increased iron uptake, demonstrated by increased serum iron and TSAT (Fig. 2 and Supplemental Fig. S2, Supplemental Digital Content, http://links.lww.com/PG9/A48). Changes in serum iron concentrations were not dose-dependent. On day 1, there was a plateauing effect between the 2 higher doses (16.6 mg b.d. and 30 mg b.d.) and on day 10, iron exposure was comparable across all doses studied. The response–time profile for TSAT was similar to that for iron.

Maltol was completely metabolized to maltol glucuronide 2.0–3.0 hours after dosing. In population pharmacokinetic analyses for maltol glucuronide, the time to C_max_ on day 1 (median: 1 hour) and day 10 (median: 0.75 hours) was similar in each dose group. C_max_ and AUC were dose-dependent (Supplemental Fig. S3, Supplemental Digital Content, http://links.lww.com/PG9/A48). Dose proportionality existed over the dose range tested, except for the predicted C_max_ on day 10.

Twenty children (54.1%) experienced a TEAE, with similar frequencies in each group (Table 2). All TEAEs were mild or moderate and all recovered or resolved. Overall, the most frequent TEAEs were gastrointestinal (overall n = 12 [32%], including fecal discoloration n = 5, diarrhea n = 3, nausea n = 2, vomiting n = 2, abdominal pain n = 1, abdominal distension n = 1, anal incontinence n = 1, constipation n = 1, and dyspepsia n = 1) and nervous system disorders (overall n = 9 [24%], headache n = 7, dizziness n = 3, lethargy n=1; Supplementary Table S2, Supplemental Digital Content, http://links.lww.com/PG9/A48). Only 1 child (in the 16.6 mg group) discontinued the study early because of a TEAE (moderate tonsillitis, not related to study drug).

**TABLE 2.** Adverse Events (Safety Population)

Patients With an AE, n (%)	Ferric Maltol 7.8 mg b.d. (n = 12)	Ferric Maltol 16.6 mg b.d. (n = 13)	Ferric Maltol 30 mg b.d. (n = 12)	Total (N = 37)
Any AE	7 (58.3)	7 (53.8)	7 (58.3)	21 (56.8)
Any TEAE	7 (58.3)	6 (46.2)	7 (58.3)	20 (54.1)
TEAE related to study drug	3 (25.0)	1 (7.7)	5 (41.7)	9 (24.3)
TEAE leading to study discontinuation	0 (0.0)	1 (7.7)	0 (0.0)	1 (2.7)
Any SAE	0 (0.0)	0 (0.0)	0 (0.0)	0 (0.0)
TEAEs by system organ class occurring in >5% of patients in any group*				
Cardiac disorders	1 (8.3)	0 (0.0)	0 (0.0)	1 (2.7)
Gastrointestinal disorders	4 (33.3)	2 (15.4)	6 (50.0)	12 (32.4)
General disorders and administration-site conditions	1 (8.3)	2 (15.4)	2 (16.7)	5 (13.5)
Infections and infestations	2 (16.7)	1 (7.7)	0 (0.0)	3 (8.1)
Injury, poisoning, and procedural complications	0 (0.0)	1 (7.7)	1 (8.3)	2 (5.4)
Neoplasms benign, malignant, and unspecified	1 (8.3)	0 (0.0)	0 (0.0)	1 (2.7)
Nervous system disorders	1 (8.3)	3 (23.1)	5 (41.7)	9 (24.3)
Respiratory, thoracic, and mediastinal disorders	3 (25.0)	0 (0.0)	1 (8.3)	4 (10.8)
Skin and subcutaneous-tissue disorders	1 (8.3)	0 (0.0)	1 (8.3)	2 (5.4)

*See Supplemental Table S1 (Supplemental Digital Content, http://links.lww.com/PG9/A48) online for a breakdown of adverse events by preferred term within each system organ class.

AE = adverse event; b.d. = twice daily; SAE = serious adverse event; TEAE = treatment-emergent adverse event.

Nine children (24%) had a TEAE related to the study drug; the most common were feces discoloration (n = 5), headache (n = 3), and dizziness, diarrhea, and fatigue (n = 2 each). Other drug-related TEAEs occurring in 1 patient each were palpitations, nausea, anal incontinence, constipation, dyspepsia, lethargy, dyspnea, and papule. There were no deaths or SAEs.

No clinically meaningful changes from baseline in vital signs or 12-lead electrocardiogram results were recorded. There were no clinically meaningful differences in mean change from baseline in hematologic or clinical chemistry measures between dose groups. Overall, individual shifts from normal to abnormal hematology and clinical chemistry laboratory results were considered not related to study drug and not clinically significant. No patients had laboratory abnormalities considered as TEAEs or SAEs.

## Discussion

In this phase 1, randomized, open-label, parallel-group study, all 3 doses of ferric maltol (7.8, 16.6, and 30 mg b.d.) increased iron uptake in children and adolescents with iron deficiency, even over the short time period studied, and were well tolerated.

Changes in serum iron and TSAT were not dose-dependent, indicating a physiologically regulated uptake of iron to meet the body’s needs. Dose proportionality existed for plasma maltol glucuronide, indicating that, as in adults, maltol is readily cleared and does not accumulate. The different pharmacokinetic profiles of iron and maltol by dose are consistent with earlier pharmacokinetic studies that showed no relationship between iron absorption and maltol metabolism (36), reflecting the body’s ability to regulate iron uptake from ferric maltol depending on physiologic need and to metabolize and eliminate unneeded maltol following dosing.

There is currently an unmet need for oral iron therapy with minimal gastrointestinal adverse effects for children with iron deficiency who are unable to tolerate oral ferrous iron compounds. Our study was not designed to confirm ferric maltol tolerability in children with iron deficiency and the short study duration may be insufficient to extrapolate to longer-term use in clinical practice; nevertheless, we believe that the reported adverse-event profile is favorable. Although TEAEs were recorded in half of our patients (potentially as a result of close monitoring), only 9 patients (24%) had TEAEs judged to be related to study drug, TEAEs were mostly mild, no patients had a severe TEAE, and all recovered or resolved at the end of the study. Only one patient discontinued treatment because of a TEAE, which was assessed as not related to the study drug.

The most common TEAEs were gastrointestinal (Table 2 and Supplemental Table S2, Supplemental Digital Content, http://links.lww.com/PG9/A48). The rate (32% overall) is consistent with data from a phase 3 trial in adults with IBD, in which the incidence of gastrointestinal TEAEs with ferric maltol was 38% (19,20). By contrast, 2 meta-analyses including several thousand patients receiving ferrous iron salts reported gastrointestinal AEs in up to 70% of cases (37,38). Gastrointestinal AEs associated with ferrous compounds can result in nonadherence in up to 50% of patients and are associated with significant treatment failure and need for follow-up investigations (38). In the current study, adherence to ferric maltol was high across the 3 doses studied, which is important for effective correction of iron deficiency (20,23,24).

The gastrointestinal AEs seen with ferrous iron preparations are likely due to the direct toxicity of unabsorbed iron on the intestinal mucosa (39). With ferric maltol, in contrast, the ferric iron remains tightly bound with maltol after oral ingestion, preventing the generation of hydroxyl radicals that can cause inflammation and gastrointestinal AEs (31,32,40). Dissociation occurs only at the point of absorption, which allows the efficient uptake of elemental ferric iron into enterocytes with relatively low daily doses without compromising efficacy (28–30). Furthermore, if absorption does not take place, the iron–maltol complex remains strongly chelated, likely reducing pathogenicity of gut microbes, given that increasing pathogen abundance is associated with free iron in the colon (21,22). Endoscopic examination was deemed unethical in this trial and we did not collect stool samples, so we cannot draw firm conclusions about the impact of ferric maltol on the gut microbiome. Nevertheless, in earlier small studies in mice and humans, ferric maltol was associated with a reduction in harmful gut bacteria (*Bacteriodites* and *Firmicutes* spp) compared with ferrous sulfate, and no cases of colitis were reported despite administration of dextran sodium sulfate to induce epithelial injury (41); these findings suggest the absence of free iron in the gut after administration of ferric maltol. Further studies should assess changes in the gut flora, including enteropathogens, and also biomarkers of intestinal inflammation before and after treatment with ferric maltol.

This phase 1 study is the first trial of ferric maltol in children and adolescents with iron deficiency, a population at increased risk of anemia (1). The stratified randomization used was effective as patient characteristics were similar in each intervention arm, allowing reliable comparison of outcomes, including safety and tolerability in the different dose groups. In addition, there was good compliance with sampling and all 37 randomized children contributed to the pharmacokinetic analysis.

Nonetheless, a limitation of our study was the relatively short period over which AEs were assessed and the small number of children in each dose group. A phase 3 trial is in development with a longer duration of treatment, a greater number of participants and a comparison group receiving ferrous iron, which will help to better characterize the AE profile.

In conclusion, in this phase 1 study, all 3 doses of ferric maltol (7.8, 16.6, and 30 mg b.d.) increased iron uptake, even over the short time period studied, and had an acceptable tolerability profile. The results from this study will help to establish a dosing schedule of ferric maltol for further investigation in larger trials of children with iron deficiency.

## Supplementary Material


