# Supplementary material for: Safety, Tolerability, and Pharmacokinetics of Oral Ferric Maltol in Children With Iron Deficiency: Phase 1 Study
Source: JPGN Rep. 2021 Jun 15;2(3):e090. doi: 10.1097/PG9.0000000000000090 (PMC10191551; doi:10.1097/PG9.0000000000000090)
Supplement: Supplementary file 1 [file pg9-2-e090-s001.pdf]

FIGURE S1. Patient disposition. b.d. = twice daily; ITT = intention-to-treat; TEAE = treatment-emergent adverse event.

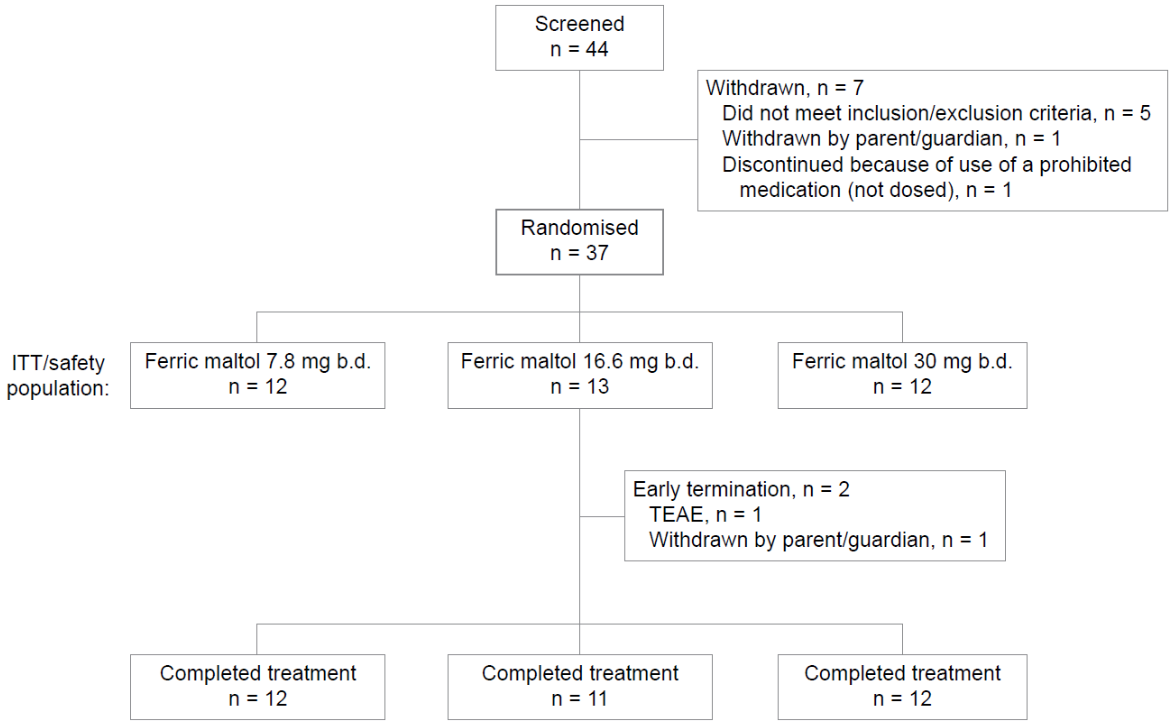

FIGURE S2. Predicted changes from baseline in (A) mean serum iron (g/mL) and (B) mean TSAT (%) by ferric maltol dose on day 1 (left panels) and day 10 (right panels) in the intention-to-treat population. b.d. = twice daily; TSAT = transferrin saturation.

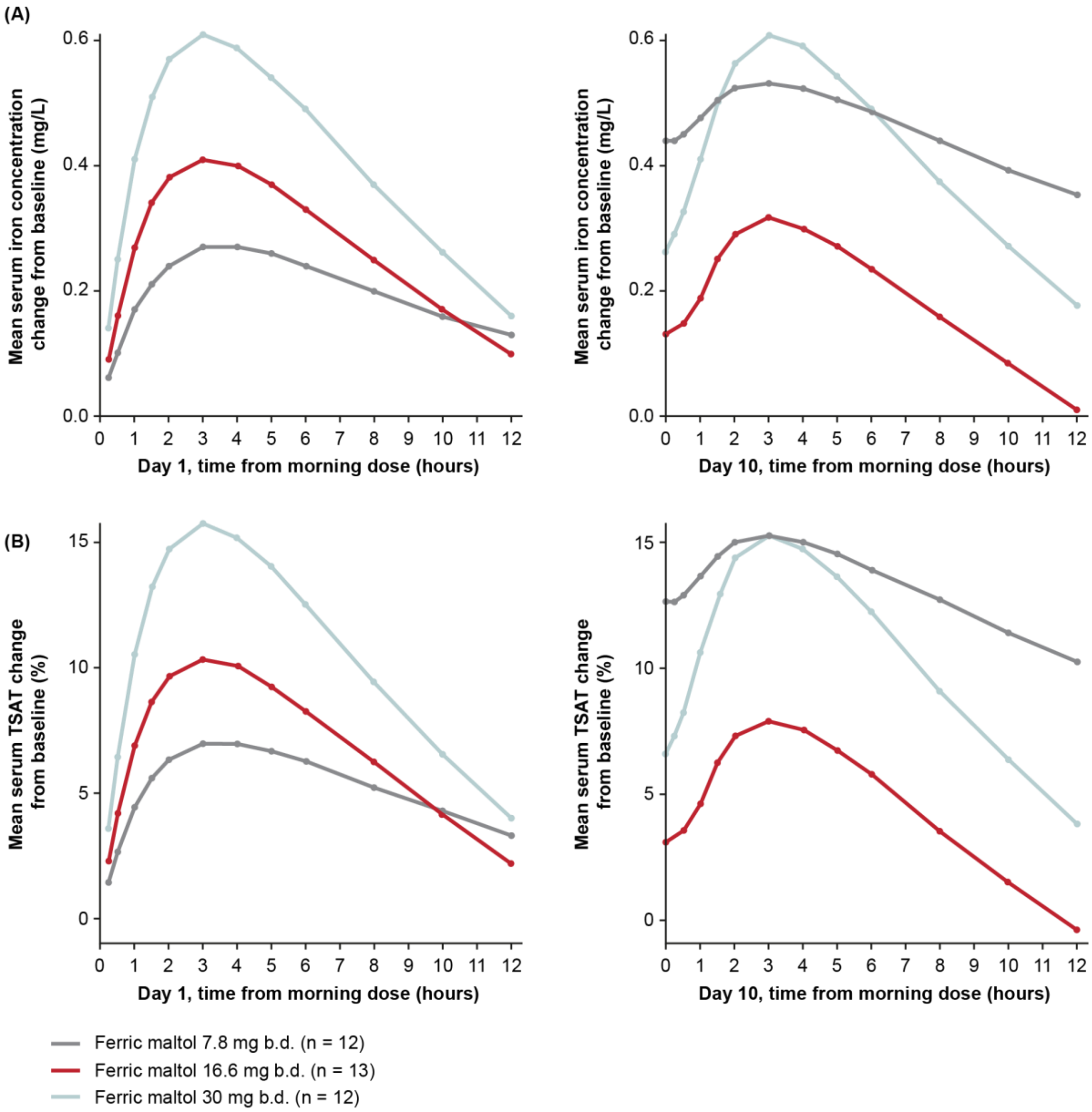

FIGURE S3. Predicted mean plasma maltol glucuronide concentrations (mg/L) by dose group on (A) day 1 and (B) day 10 on linear scales (left panels) and semi-logarithmic scales (right panels) in the intention-to-treat population. b.d. = twice daily.

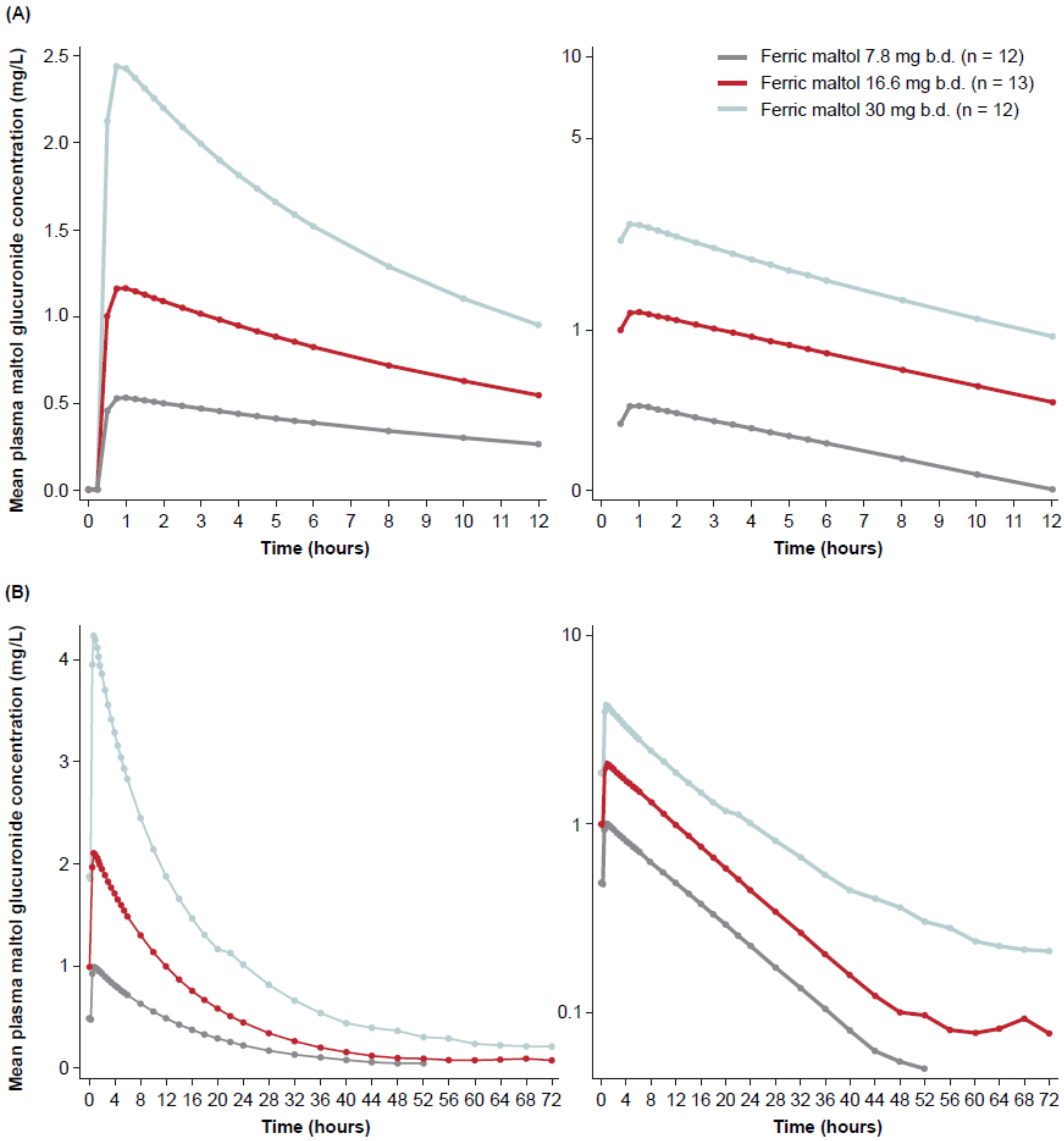

TABLE S1. Recruitment of patients at each dose level by site

Site	Ferric maltol 7.8 mg b.d. (n = 12)	Ferric maltol 16.6 mg b.d. (n = 13)	Ferric maltol 30 mg b.d. (n = 12)	Total (N = 37)
1	3	6	1	10
2	5	2	1	8
3	1	3	4	8
4	3	2	2	7
5			3	3
6			1	1

TABLE S2. Most frequent TEAEs (affecting >5% in any group) by system organ class and preferred term

Patients with a TEAE, n (%)	Ferric maltol 7.8 mg b.d. (n = 12)	Ferric maltol 16.6 mg b.d. (n = 13)	Ferric maltol 30 mg b.d. (n = 12)	Total (N = 37)
Cardiac disorders	1 (8.3)	0 (0.0)	0 (0.0)	1 (2.7)
Palpitations	1 (8.3)	0 (0.0)	0 (0.0)	1 (2.7)
Gastrointestinal disorders	4 (33.3)	2 (15.4)	6 (50.0)	12 (32.4)
Feces discolored	1 (8.3)	1 (7.7)	3 (25.0)	5 (13.5)
Diarrhea	2 (16.7)	0 (0.0)	1 (8.3)	3 (8.1)
Nausea	1 (8.3)	0 (0.0)	1 (8.3)	2 (5.4)
Vomiting	1 (8.3)	1 (7.7)	0 (0.0)	2 (5.4)
Abdominal distension	0 (0.0)	0 (0.0)	1 (8.3)	1 (2.7)
Abdominal pain	0 (0.0)	0 (0.0)	1 (8.3)	1 (2.7)
Anal incontinence	0 (0.0)	0 (0.0)	1 (8.3)	1 (2.7)
Constipation	0 (0.0)	0 (0.0)	1 (8.3)	1 (2.7)
Dyspepsia	0 (0.0)	0 (0.0)	1 (8.3)	1 (2.7)
Lip dry	0 (0.0)	0 (0.0)	1 (8.3)	1 (2.7)
General disorders and administration-site conditions	1 (8.3)	2 (15.4)	2 (16.7)	5 (13.5)
Fatigue	1 (8.3)	1 (7.7)	2 (16.7)	4 (10.8)
Injection-site pain	0 (0.0)	1 (7.7)	0 (0.0)	1 (2.7)
Pyrexia	0 (0.0)	0 (0.0)	1 (8.3)	1 (2.7)
Infections and infestations	2 (16.7)	1 (7.7)	0 (0.0)	3 (8.1)

Cellulitis	1 (8.3)	0 (0.0)	0 (0.0)	1 (2.7)
Nasopharyngitis	1 (8.3)	0 (0.0)	0 (0.0)	1 (2.7)
Tonsillitis	0 (0.0)	1 (7.7)	0 (0.0)	1 (2.7)
Injury, poisoning, and procedural complications	0 (0.0)	1 (7.7)	1 (8.3)	2 (5.4)
Ligament sprain	0 (0.0)	0 (0.0)	1 (8.3)	1 (2.7)
Skin abrasion	0 (0.0)	1 (7.7)	0 (0.0)	1 (2.7)
Neoplasms benign, malignant, and unspecified	1 (8.3)	0 (0.0)	0 (0.0)	1 (2.7)
Nevus hemorrhage	1 (8.3)	0 (0.0)	0 (0.0)	1 (2.7)
Nervous system disorders	1 (8.3)	3 (23.1)	5 (41.7)	9 (24.3)
Headache	1 (8.3)	2 (15.4)	4 (33.3)	7 (18.9)
Dizziness	0 (0.0)	1 (7.7)	2 (16.7)	3 (8.1)
Lethargy	0 (0.0)	0 (0.0)	1 (8.3)	1 (2.7)
Respiratory, thoracic, and mediastinal disorders	3 (25.0)	0 (0.0)	1 (8.3)	4 (10.8)
Cough	1 (8.3)	0 (0.0)	1 (8.3)	2 (5.4)
Dyspnea	1 (8.3)	0 (0.0)	0 (0.0)	1 (2.7)
Nasal congestion	0 (0.0)	0 (0.0)	1 (8.3)	1 (2.7)
Sneezing	1 (8.3)	0 (0.0)	0 (0.0)	1 (2.7)
Skin and subcutaneous-tissue disorders	1 (8.3)	0 (0.0)	1 (8.3)	2 (5.4)
Granuloma skin	1 (8.3)	0 (0.0)	0 (0.0)	1 (2.7)
Papule	0 (0.0)	0 (0.0)	1 (8.3)	1 (2.7)

b.d. = twice daily; TEAE = treatment-emergent adverse event.
